# Multi-Hollow Surface Dielectric Barrier Discharge for Bacterial Biofilm Decontamination

**DOI:** 10.3390/molecules26040910

**Published:** 2021-02-09

**Authors:** Zlata Kelar Tučeková, Lukáš Vacek, Richard Krumpolec, Jakub Kelar, Miroslav Zemánek, Mirko Černák, Filip Růžička

**Affiliations:** 1 Department of Physical Electronics, Faculty of Science, Masaryk University, Kotlářská 2, 611 37 Brno, Czech Republic; krumpolec@mail.muni.cz (R.K.); jakub.kelar@mail.muni.cz (J.K.); mzemanek@mail.muni.cz (M.Z.); 54782@mail.muni.cz (M.Č.); 2 The Department of Microbiology, Faculty of Medicine, Masaryk University, St. Anne’s University Hospital, Pekařská 53, 602 00 Brno, Czech Republic; 258662@mail.muni.cz (L.V.); fruzic@fnusa.cz (F.R.)

**Keywords:** atmospheric pressure plasma, low-temperature plasma, plasma-activated media, bacterial biofilm, decontamination

## Abstract

The plasma-activated gas is capable of decontaminating surfaces of different materials in remote distances. The effect of plasma-activated water vapor on *Staphylococcus epidermidis*, methicillin-resistant *Staphylococcus aureus*, *Pseudomonas aeruginosa*, and *Escherichia coli* biofilm contamination was investigated on the polypropylene nonwoven textile surface. The robust and technically simple multi-hollow surface dielectric barrier discharge was used as a low-temperature atmospheric plasma source to activate the water-based medium. The germicidal efficiency of short and long-time exposure to plasma-activated water vapor was evaluated by standard microbiological cultivation and fluorescence analysis using a fluorescence multiwell plate reader. The test was repeated in different distances of the contaminated polypropylene nonwoven sample from the surface of the plasma source. The detection of reactive species in plasma-activated gas flow and condensed activated vapor, and thermal and electrical properties of the used plasma source, were measured. The bacterial biofilm decontamination efficiency increased with the exposure time and the plasma source power input. The log reduction of viable biofilm units decreased with the increasing distance from the dielectric surface.

## 1. Introduction

The low-temperature plasma (LTP) generated by various dielectric barrier discharges (DBD) at atmospheric pressure is widely investigated to generate reactive species for surface modification and decontamination. The hydroxyl (OH) radical belongs to the essential reactive agents applied for the generation of OH functional groups on the treated surface and hydrogen peroxide (H_2_O_2_) in plasma products [1,2]. Such products can enhance biocompatibility and nanoparticle immobilization, and clean and decontaminate various surfaces [3,4,5]. Currently, the novel approaches using LTPs are studied to prepare self-cleaning, antibacterial, and biocompatible substrates, especially textiles with smart coatings [6,7,8,9].

The OH radical itself directly impacts microorganism cell membranes and provides inactivation of various pathogens [10,11] due to its highest oxidation potential [12]. Various LTPs have been developed to generate OH radicals by water molecule dissociation. However, many of them work at low pressures where stable and “cold” LTPs are easy to generate and sustain [13,14,15].

Plasma generation of OH radicals that can be carried out at atmospheric pressure and preferably using humid air would be much more practical owing to the cost reduction of the apparatus not requiring vacuum equipment for the process and the possibility of large-scale treatments. Humid air is often used to enhance the effect of plasma-activated gaseous media and the generation of OH radicals [16,17]. However, when operated in humid air, DBDs plasmas generate also significant amounts of toxic ozone, and nitric oxide and nitrogen dioxide [18], particularly when the air contains less than 50% of water vapor. As discussed in the literature [19,20], if more water vapor is added to the air, the generation of OH radicals can be much more complicated [19,20,21] since the high water vapor concentrations increase the LTP generation voltage or even prevent plasma generation due to condensation [20]. Thus, the effective discharge and humid gas feeding design are crucial for the reliability of the plasma source in a potential application.

As a solution to this problem, a vast range of approaches was studied to achieve the generation of stable LTP in high-humidity air or water vapor at atmospheric pressure, such as admixtures of expensive noble gasses (helium or argon) in the plasma working gas [19,22] and alternative design of the discharge configurations [23] or pulsating DC power to excite the air-water plasma [24].

Multi-hollow surface dielectric barrier discharge (MSDBD) has been successfully tested and investigated for plasma treatment of materials like glass and silicon [25], polycarbonate polymer [26], and food processing [27,28]. This ambient air plasma source offers the capability of a remote plasma modification or decontamination of materials at enlarged distances [18,29].

The electrical characteristics and temperature measurements of MSDBD in different gases and at a wide gas flow range [25,26,27,28] were essential to understanding its safe operation and potential application. The main advantage of the robust MSDBD electrode system is its capability to withstand high-temperature loads. The MSDBD can generate ambient air plasma at a flow rate of 1 L/min and input power 30 W while heated up to surface temperature of almost 250 °C for a long time [25]. This laid the groundwork to validate challenging requirements presented in this study.

We developed a proprietary device and method for plasma activation of flowing gas mixtures with high water vapor concentrations based on the modified MSDBD plasma technology [30]. This device enables plasmachemical splitting of pure water vapor molecules for the generation of OH radicals and their products, e.g., H_2_O_2_, without stabilizing gas admixtures and toxic byproducts. Additionally, it is simple in design, easy to operate and not too expensive. The decontamination of bacterial biofilm in remote distances was investigated to prove the high efficiency of generated plasma-activated water vapor (PAWV) and water aerosol in remote distances (PAWA). The properties and decontamination efficiency of the plasma source generating PAWV and PAWA are presented in this study.

## 2. Results and Discussion

### 2.1. Characterization of the MSDBD Device for Plasma-Activated Media Generation

**Figure 1:**
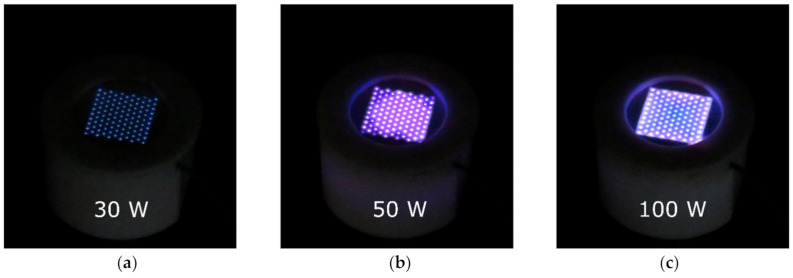
The photographs of multi-hollow surface dielectric barrier discharge (MSDBD) at the stabilized flow of pure water vapor at different input power (**a**–**c**).

**Figure 2:**
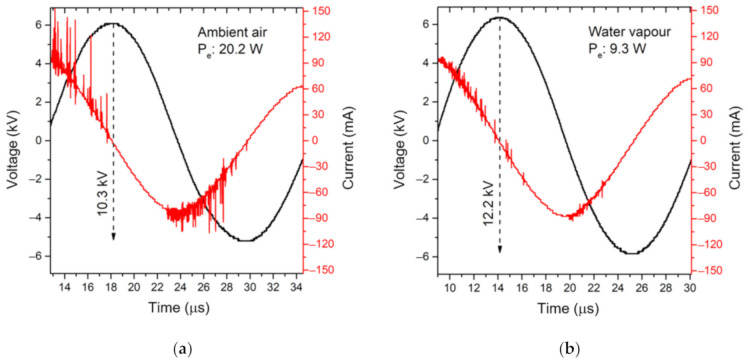
The voltage and current waveforms of MSDBD in ambient air (30 W) (**a**) and water vapor (26 W) (**b**).

The experiments reported in this study were performed at the stabilized flow of pure water vapor through the MSDBD electrode system. The flow was regulated to 0 L/min in ambient air (heating of the ceramics) and approximately 25 L/min of water vapor during measurements. The photographs of MSDBD in water vapor at different input power are shown in Figure 1. The setting of the non-stabilized AC high voltage (HV) power supply initial power was fixed, and it caused the change of the energy transfer (and actual efficiency) into the electrode system in the atmosphere of water vapor, shown in Figure 2. 

Due to the change of MSDBD properties in the atmosphere with high humidity, we can observe alteration of peak-to-peak voltage and discharge current pulses. These correlate with the increased ignition voltage and higher requirements for effective energy transfer to the discharge. The consequent effective power (*P_e_*) fed to the electrode system decreased in the pure water vapor. The plasma power density in ambient air and water vapor was estimated to be 6.2 and 2.9 W/cm^2^.

**Figure 3:**
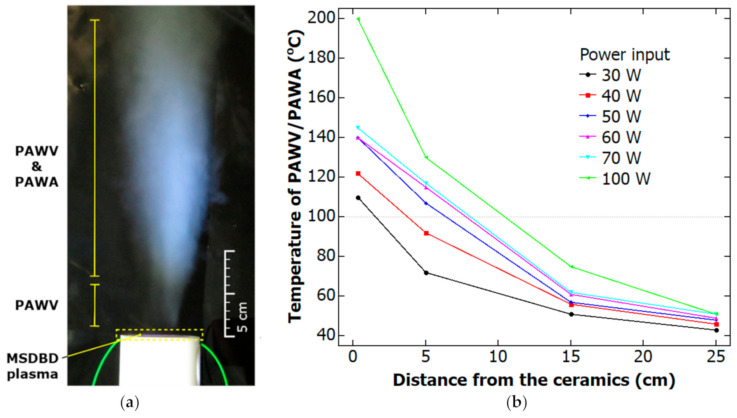
The photograph of MSDBD at the stabilized flow of pure water vapor used for bacteria decontamination (**a**) and the graph of temperature in dependence of distance from ceramics and input power (**b**).

The ceramic surface temperature was measured in the input power’s dependence at a constant water vapor flow (25 L/min). The ceramic surface temperature varied from 107 °C at 30 W to 216 °C at 100 W of input power (Figure 3). This dependence within the mentioned range can be interpreted as a linear function. This correlates with the estimated efficiency of the power supply and energy dissipation when the MSDBD operates at the constant water vapor flow.

### 2.2. Characterization of Generated Plasma-Activated Media

The temperatures in different remote distances (Figure 3) used for biofilm decontamination were measured for plasma-activated and not-activated water vapor. The temperatures were the same within the measurement error for plasma-activated and non-activated media. The temperatures were estimated to 46, 38, and 37 °C in the distance 20, 30, and 40 cm. The average increase in energy consumption during the temperature measurement and decontamination process was 1% due to the MSDBD plasma-activation of water vapor at 30 W power input.

The pH of condensed water after the plasma activation was in the range of 6–7. Semiquantitative test strips evaluated the relative concentration of active species in the condensed plasma-activated media. The ammonium and formaldehyde were not detected in PAWA/PAWV by used test strips.

The peroxide concentration in condensed PAWV/PAWA was estimated to be in the range of 120–150 mg/L. We obtained a relatively high concentration in condensed plasma-activated media. The energy yield for hydrogen peroxide generation was estimated from *P_e_* to approximately 100 g/kWh. The correct evaluation requires precise knowledge of power transferred into the discharge (not the electrode system) [31]. The presented value is only estimative and does not reflect the in situ measurement of hydrogen peroxide production. The most probable hydrogen peroxide production is water molecule splitting, OH radical reactions during the plasma activation, and the preservation by effective absorption into the water [1,2,32].

The nitrate and nitrite concentrations were estimated to be approximately 50 mg/L and 1 mg/L, respectively. The nitrogen species originated from the MSDBD plasma in ambient air present in the provisional chamber. The ozone test strips confirmed the ozone production in ambient air while heating the ceramics. The strip exceeded its detection limit after a few seconds of MSDBD operation. The preservation of the ozone and its dilution to the condensed PAWV/PAWA is highly improbable, as the temperature of the plasma-activated media and atmosphere in the chamber increases the reaction rates for direct ozone decomposition processes [31,33].

The pH of used distilled water and condensed non-activated water vapor was approximately 7. The active species were not detected in condensed non-activated water vapor. 

### 2.3. Bacterial Biofilm Decontamination

The adjustment of the process and preliminary experiments of bacterial inactivation in the biofilm were conducted on *E. coli*. This bacteria species proved to be very sensitive to increased temperature. Thus, the results are overestimated and accompanied by a significant error. The maximum and minimum reduction in bacteria numbers of 4.8 log and 2.9 logs were achieved at 20 cm (150 s) and 40 cm (30 s) respectively, revealing an obvious decrease of log reduction with increased distance from the plasma source and decreased exposure time. These values were obtained by the standard CFU plate count only.

The *E. coli* and most clinically relevant non-sporulating bacteria are often thermally inactivated at about 50 °C and above. The lethal effect of temperature grows stronger with prolonged periods, and the temperature rise [34,35,36]. The temperature of the polypropylene nonwoven textile (PP-NT) coupon was measured at 20 cm. The temperature was 48 °C after 30 s and slowly increased with treatment time. The maximum temperature of 53 °C was measured on the surface with a film of condensed media after 150 s, while the PP-NT coupon kept lower temperature.

The experiments with *E. coli* bacterial biofilm initiated serial tests of bacteria extraction from the PP-NT coupon, efficient separation and dispersion using ultrasound sonication, and improved LOG reduction evaluation by start of growth time (SGT) quantification. The SGT method utilizes a 24-h-long measurement of the optical density of the bacterial sample growth after the extraction and separation process. The SGT method used the calibration curve based on the known samples of bacterial suspensions to determine bacterial densities of unknown samples. The SGT method’s main advantage was reducing the time and material (agar, Petri dish) necessary to perform the CFU plate count method.

**Figure 4:**
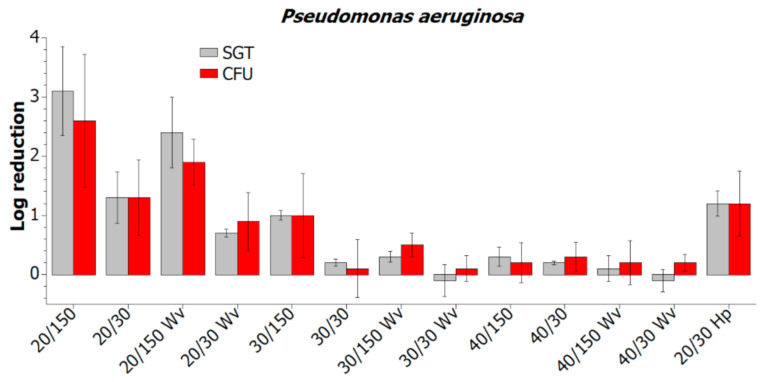
Comparison of decontamination efficiency of plasma-activated and not-activated water vapor (“Wv”) in remote distances evaluated by the SGT and CFU method.

**Figure 5:**
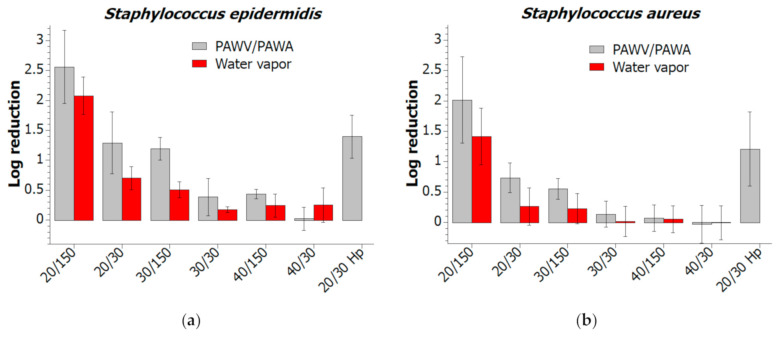
Decontamination efficiency of plasma-activated and not-activated water vapor (“s”) in remote distances tested for *S. epidermidis* (**a**) and *S. aureus* (**b**).

The decontamination of *P. aeruginosa* biofilm (Figure 4) was improved by plasma activation of water vapor when applied in the distance of 20 and 30 cm. The maximum log reduction of 3.1 was achieved at 20 cm by the 150 s treatment. Similar tendencies were observed for *S. aureus* and *S. epidermidis* (Figure 5) with a maximum log reduction of 2.0 and 2.6, respectively. 

Compared to pure water vapor decontamination results (labeled with “Wv”), the log reduction was higher by the value of 0.6 on average. These values were obtained by both the SGT method and the standard CFU plate count.

The plasma-activated water vapor was applied at a distance of 20 cm for 30 s with the power increased to 150% to test the effect of MSDBD input power (labeled as “20/30 Hp”). This effect can be observed only in *S. aureus*, where log reduction increased by 0.5. The increase of decontamination efficiency for *S. epidermidis* and *P. aeruginosa* was insignificant. These results are accompanied by a relatively high deviation, common for standard bacterial cell counts. However, the decontamination effect of PAWA and PAWV in remote distances is unique and can be improved by prolonged treatment or direct exposure to plasma-activated media.

The inactivation of bacteria is dependent on biofilm thickness, the penetration depth of active particles and induced secondary products. Thus, hydrogen peroxide and OH radicals are very important in the process [2,11,37]. To test the effect of plasma-activated media compared to water vapor, we exposed the *S. aureus* biofilm to condensates and physiological solution (control sample). The PP-NT coupons were immersed in these solutions for 30 and 60 min. The bacterial population in condensed water vapor (after 30 min) decreased by 0.03 Log compared to the control sample. In condensed PAWV/PAWA, the bacterial population decreased by 0.70 Log (after 30) and by 1.39 Log (after 60 min). The hydrogen peroxide and active species in PAWV/PAWA were active within a long time and ensured the bacterial decontamination in biofilm. These results were obtained by the SGT method.

The LTPs provide the generation of high energetic particles within discharge channels or in their vicinity. These particles (electrons, ions, and excited and metastable particles) and UV radiation directly impact cell membranes and chemical reactions in plasma gas [16,38,39,40]. However, the region for effective decontamination (or distance from discharge) is often limited. The active particles generated in plasma-activated media and plasma afterglow can provide the oxidative effects directly associated with microbial inactivation [12]. The highest oxidation potential of OH radical plays an important role in such an inactivation process. Thus, the generation of LTP in water vapor containing atmosphere represents its significant source.

In practice, it turned out that humid air is a very convenient discharge atmosphere for surface decontamination [16,41,42,43,44,45,46,47,48]. Given the extensive studies available, the literature on the subject is surprisingly internally inconsistent [49], apparently also due to the complexity of the humid air plasma chemistry. Eto et al. [50] argued that OH radicals, produced in humid air by a DBD through direct chemical reactions with water molecules, plays the main role in the inactivation of *Geobacillus stearothermophilus* spores. Kang et al. [51] investigated the efficiency of OH radical production in the air by pulsed discharges and found that the efficiency of the OH radical generation in atmospheric air depends on its relative humidity and has a maximum at 55%. Han et al. [52] reported as the main reactive species in humid air LTP are atomic and singlet oxygen, nitric oxides, ozone, H_2_O_2_, OH radical, and HNO_x_. Sasaki et al. [53] stated in the air with high relative humidity (RH up to 70%), OH radical quickly takes part in chemical reactions (half-period life in biological cells 1 ns) and generates a significant amount of peroxides contributing to the inactivation of *Bacillus atrophaeus* spores.

The ability to inactivate planktonic bacteria by LTP activated water vapor and water was tested for several applications. The inactivation effect of active particles in the activated water can persist for a very long time (several weeks), and it was reviewed in [37,54,55]. The decontamination of biofilm by activated water or condensate can be employed. However, the efficient preparation of active particles with a disinfectant effect on biofilm is needed [56,57,58]. For example, the gas phase of nitric oxides, ozone, and H_2_O_2_ can be dissolved or generated as a gas radical/atom reaction with a radical/atom at the liquid interface [1,38,59]. The pathways of active species generation in the gas and liquid phase, and the resistance and survival of biofilm exposed to plasma-activated water, are reviewed in [55].

PAWV/PAWA generation’s potential applications are decontamination, hands and skin sanitization, deodorization, detoxification, etching, and activated water generation. Currently, we are working on the first implementation of the technology. The plasma device is being tested for utilization in a decontamination/sterilization chamber. The improved inactivation effect is ensured by prolonged PAWV/PAWA generation (up to 100 min).

## 3. Materials and Methods

### 3.1. Plasma-Activated Media Generation

#### 3.1.1. Device for the Generation of Plasma-Activated Media

The device for the PAWV/PAWA generation used in this work was built as described and illustrated in Figure 3 in the patent application EP3585136A1 [30]. The device consisted of an MSDBD electrode system that was connected to the water steam generator. The MSDBD electrode system (Kyocera Inc., Kyoto, Japan) was created by two parallel metallic electrodes embedded in alumina ceramics and was powered by an AC-HV power supply. The interelectrode distance was 0.5 mm, and the total thickness of the ceramic body was 1.6 mm. The MSDBD unit features 105 holes, of diameter 0.6 mm, evenly distributed over the area 18 × 18 mm^2^, allowing the flow of working gas mixture through the ceramic body and active plasma area [26]. Compact steam boiler (250 mL standard steam boiler installed, e.g., in commercial hand-held steam cleaners) of maximal power 1 kW (Silvercrest, Bochum, Germany) was used as a steam generator. A precise needle valve controlled the flow of water vapor from the generator. The device was equipped with a custom temperature control system and water condensate removal system. The generated PAWV was fed to the open ambient air.

#### 3.1.2. Electrical Measurements

The properties of the MSDBD plasma source in ambient air and water vapor were obtained at maximal measured power used for biofilm decontamination. The input power and power consumption were measured by wattmeter Voltcraft Energy Monitor 3000 (Conrad Electronic International, Hirschau, Germany). The input power changed after the water vapor flow due to the use of a non-stabilized AC-HV power supply. The power consumption was repeatedly measured for short (30 s) periods during the continuous water vapor generation (250 mL). The increase in energy required was calculated using average consumption values measured with and without plasma.

**Figure 6:**
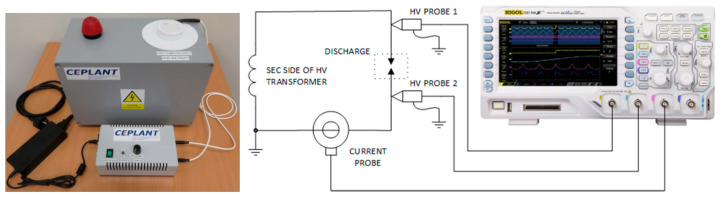
The photograph of the device for plasma-activated water vapor (PAWV)/plasma-activated water aerosol (PAWA) generation and electrical measurement schematics.

To capture actual sinusoidal (25–27 kHz) high-voltage and discharge current (Figure 6), we used the four-channel oscilloscope Rigol DS1104Z-S Plus (Rigol Technologies, Beijing, China). The voltage waveform was estimated as the subtraction of voltage signals measured on both electrodes by (1:1000) high voltage probes P6015A (Tektronix, Beaverton, OR, USA). The current waveform was measured by standard Pearson current monitor model 4100 (Pearson Electronic, Palo Alto, CA, USA). The effective power was calculated as the direct integration of voltage and current product. The plasma power density was then estimated from *P_e_* supplied to the MSDBD electrode system.

#### 3.1.3. Temperature Measurements

**Figure 7:**
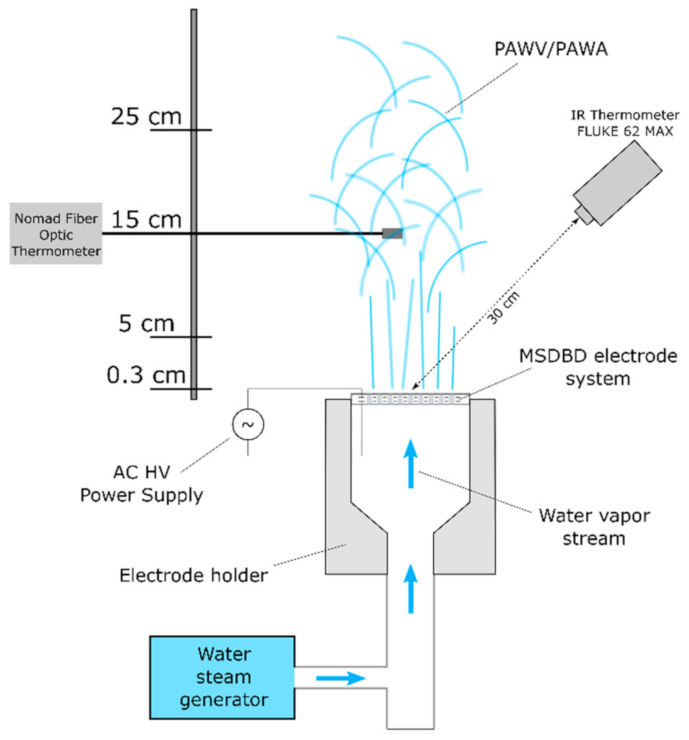
The schematics of the device for PAWV/PAWA generation and temperature measurements.

PAWV/PAWA temperature was measured using Nomad Fiber Optic Thermometer NMD (Neoptix, Quebec, QC, Canada) with Fiber Optic Temperature Sensor T1S-02-WNO-PT05 enclosed in a copper casing. The micro-hollow electrode’s ceramic surface temperature was measured using the IR thermometer Fluke 62 MAX (Fluke, Everett, WA, USA). The emissivity of the used alumina was set to 0.9. The sensor was placed 30 cm from the ceramics at the angle of 30°, as shown in Figure 7.

#### 3.1.4. Characterization of Plasma-Activated Media

The relative concentration of active species in the condensed PAWV/PAWA was evaluated by semiquantitative test strips Quantofix (Macherey-Nagel, Düren, Germany). The detected species (detection limits) were ammonium (0–400 mg/L), formaldehyde (0–200 mg/L), peroxide (0–100 mg/L), nitrate, and nitrite (0–500 mg/L and 0–80 mg/L). To confirm the ozone production in the MSDBD ambient air plasma, we also used the semiquantitative strips Ozone Test (0–210 μg/m^3^, Macherey-Nagel, Düren, Germany). The pH of the condensed PAWV/PAWA was measured by universal pH indicator paper (pH 0–12, Lach-ner, Neratovice, Czech Republic). The PAWV/PAWA condensation on provisional chamber walls was ensured using a standard laboratory beaker (5 L, Simax, Sázava, Czech Republic). To decrease the tested liquid temperature and for result confirmation, each measurement was repeated for condensate diluted by a factor of 2.

### 3.2. Decontamination of Bacterial Biofilms

#### 3.2.1. Bacterial Biofilm Preparation

Bacterial strains *Staphylococcus epidermidis* (CCM 7221), methicillin-resistant *Staphylococcus aureus* (CCM 4750 = ATCC 43300), and *Escherichia coli* (CCM 3988 = ATCC 10536) were obtained from the Czech Collection of Microorganisms (Masaryk University, Brno, Czech Republic). The *Pseudomonas aeruginosa* (FB 45) strain was obtained from the Collection of Microorganisms of St. Anne’s University Hospital in Brno (Czech Republic). Bacteria were grown in the brain heart infusion broth (*S. aureus*, *S. epidermidis*, and *E. coli*) or tryptic soy broth (*P. aeruginosa*) (Thermo Fisher Scientific Inc., Oxoid, UK). Single-species bacterial biofilms were grown on PP-NT coupons with a diameter of 1 cm for further decontamination by MSDBD generated PAWV/PAWA. Briefly, overnight bacterial culture was inoculated in a fresh medium and grown to the optical density of 0.5 McFarland standard (ca. 10^8^ CFU/mL). Then, bacterial suspensions were diluted 100 times with fresh medium supporting biofilm formation (BHI or TSB medium supplemented with 1% glucose). Biofilm was formed during cultivation at 37 °C for 20 h.

#### 3.2.2. Decontamination Effect Evaluation

The biofilm samples were rinsed twice with PBS and then exposed to plasma-activated water vapor and pure water vapor in distances of 20, 30, and 40 cm for 30 or 150 s. After the treatment, samples were submerged in the phosphate-buffered saline (PBS), resazurin (Sigma-Aldrich, St. Louis, MO, USA) was added to the final concentration of 5 µg/mL and incubated for 2 h at 37 °C.

The decontamination effect was evaluated firstly by a cell viability assay based on the resazurin fluorescence measurement analysis using multiwell plate reader Tecan Infinite M200 PRO (Tecan Trading AG, ZÜRICH, Switzerland). Further, to separate bacteria from PP-NT coupons and to disperse them into the individual bacteria cells, samples were sonicated twice for one minute (0.5 W/cm^2^) in ultrasonic bath Sonorex Digiplus DL 102 H (Bandelin, Berlin, Germany) and thoroughly mixed by pipetting.

Further, individual bacterial suspensions were diluted and plated, and CFU/mL counts were determined. In addition, the adjusted SGT method for high throughput quantification (according to [60]) was employed. This method used three main approaches to count bacteria in the standard calibration suspension. The simplest one used the determination of the most probable number (statistical method). The number of bacteria in the suspension was also determined by standard CFU plate counting.

## 4. Conclusions

The presented work investigated the performance of the proprietary device and its capability to generate plasma-activated media with high humidity for biofilm decontamination. The electrical properties and temperature of MSDBD for PAWV/PAWA generation were measured in challenging conditions of high humidity and temperature. The power supply’s energy efficiency decreased in the presence of water vapor. The ceramics’ temperature allowed the PAWV/PAWA generation without the water vapor condensation on the MSDBD electrode system at 30 W of input power.

The properties of PAWV/PAWA were measured after its condensation. The concentration of hydrogen peroxide was approximately 120–150 mg/L. The concentrations of nitrate and nitrite were 50 and 1 mg/L, respectively. The ozone in the condensate was not measured. However, we expected its insignificant preservation. The nitrate, nitrite, and ozone originated from the ambient air MSDBD plasma generated prior to the water vapor flow.

The PAWV/PAWA proved to have a biocidal effect on *E. coli*, *P. aeruginosa*, *S. aureus*, and *S. epidermidis* biofilm. To estimate the log reduction of viable bacteria in biofilm, the novel SGT method for high throughput quantification was tested compared to standard CFU plate counting. This method allowed the decrease of result deviations. In comparison to water vapor, the decontamination by PAWV/PAWA resulted in an approximately 0.5 log reduction increase. The decontamination by PAWV/PAWA was more effective in shorter distances from the MSDBD electrode system, with increased treatment time and increased input power.

The proprietary device introduces the novel method of biocidal active species generation in a high humidity atmosphere. The device generates the plasma-activated water vapor and aerosol, which provide preservation of soluble active particles and transport to larger volume or inaccessible space (e.g., capillaries, cavities, corners, etc.). The high performance of the used MSDBD electrode system presents a solution to in situ hydrogen peroxide production without drawbacks such as low pressure, noble gases, or special power supply of discharge configuration.

## 5. Patents

The patents resulting from the work reported in this manuscript are European patent EP3585136A1 “A method and device for generating low-temperature electrical water-based plasma at near-atmospheric pressures and its use” and Czech utility model CZ33565U1 “Device for generating plasma-activated vapor or aerosol using a flowing gas with a significant proportion of water vapor”.

## Data Availability

The part of the data presented in this study are openly available in [Google Patents] at [https://patents.google.com/patent/EP3585136A1/en?oq=EP3585136A1 (accessed on 8 February 2021)], reference number [30].

## Sample Availability

Samples of the compounds are not available from the authors.
